# Exercise during chemotherapy or chemoradiotherapy and treatment delivery and tumor response outcomes: a scoping review

**DOI:** 10.1186/s12885-026-15992-6

**Published:** 2026-04-24

**Authors:** Takuya Fukushima, Jiro Nakano, Katsuyoshi Suzuki, Keiichi Osaki, Takashi Tanaka, Taro Okayama, Junichiro Inoue, Jack B. Fu, Shinichiro Morishita

**Affiliations:** 1 Faculty of Rehabilitation, Kansai Medical University, Osaka, Japan; 2 Division of Rehabilitation Medicine, Shizuoka Cancer Center, Shizuoka, Japan; 3 Department of Rehabilitation, Panasonic Health Insurance Organization, Matsushita Memorial Hospital, Osaka, Japan; 4 Department of Physiotherapy, Faculty of Medical Care and Nursing, Osaka University of Medical Sciences, Osaka, Japan; 5 Division of Rehabilitation Medicine, Kobe University Hospital, Kobe, Japan; 6 University of Texas MD Anderson Cancer Center, Houston, TX USA; 7 Department of Physical Therapy, School of Health Science, Fukushima Medical University, 10-6, Sakaemachi, Fukushima, Fukushima 960-8516 Japan

**Keywords:** Cancer, Relative dose intensity, Treatment completion, Tumor response, Exercise oncology

## Abstract

**Background:**

Exercise during cancer treatment has been proposed not only as supportive care but also as a biologically active intervention with potential systemic effects. Mechanistic studies suggest that exercise modulates immune function and induces systemic signaling through exerkines, which may influence responsiveness to cancer therapies. However, evidence regarding exercise effects on treatment delivery, such as relative dose intensity, treatment completion, and tumor response, remains limited and heterogeneous. This scoping review aimed to map and summarize existing literature on associations between exercise, treatment delivery, and tumor response outcomes, highlighting patterns and gaps that inform future research.

**Methods:**

We searched the PubMed/MEDLINE, Cochrane Library, and Cumulative Index to Nursing and Allied Health Literature Complete databases from inception to November 6, 2025. Studies examining the association between exercise interventions and treatment-related outcomes in patients with cancer were included.

**Results:**

Following eligibility assessment, 23 studies comprising 31 exercise interventions were included. Most studies have focused on breast and colorectal cancers and spanned various treatment phases, ranging from neoadjuvant to adjuvant settings. Treatment-related outcomes most commonly reflected treatment delivery and tolerance (e.g., relative dose intensity, dose delay or reduction, and treatment completion). Moreover, tumor response outcomes were reported, including pathological complete response, tumor size, response according to RECIST, and downstaging. Exercise interventions commonly consisted of aerobic exercise alone or in combination with resistance training, were predominantly supervised, and were often prescribed at moderate-to-high intensities, with a frequency of three to four sessions per week. Associations with favorable treatment-related outcomes were more frequently reported for supervised, moderate-to-high intensity exercise; however, null findings were common across all outcomes and cancer types.

**Conclusions:**

Current evidence indicates an inconsistent association between exercise and selected treatment delivery and tumor response outcomes in patients with cancer, suggesting a potential, context-dependent role of exercise under specific conditions. Future studies should clearly differentiate among treatment tolerance, delivery, and tumor response outcomes and investigate whether—and how—exercise influences these outcomes across various cancer types and treatment phases.

**Supplementary Information:**

The online version contains supplementary material available at 10.1186/s12885-026-15992-6.

## Background

Maintaining treatment efficacy during cancer therapy is critical for achieving favorable oncologic outcomes. Reduced treatment completion rates and delayed and reduced doses, commonly summarized as relative dose intensity (RDI), have been consistently associated with poorer survival and disease control across multiple cancer types [1]. Therefore, identifying supportive interventions that enable patients to tolerate and complete their planned cancer treatment has become an important clinical priority.

Exercise is a promising additional treatment for cancer. Rather than being conceptualized solely as supportive care or rehabilitation, exercise is increasingly recognized as a biologically active cancer therapy with the potential to modify host–tumor interactions. Courneya and Booth proposed a clinical oncology framework that positions exercise as an adjunct cancer treatment capable of influencing treatment tolerance, tumor biology, and clinical outcomes [2]. Furthermore, recent translational initiatives, such as the Moving Through Cancer framework led by Schmitz and Rogers, emphasize immune regulation and systemic signaling as key mechanisms underlying the therapeutic potential of exercise [3]. Accumulating evidence at the biological level suggests that exercise-induced secreted factors ("exerkines") may modulate inflammation, immune activity, and the tumor microenvironment. This provides biological plausibility for exercise–treatment interactions [4]. These conceptual and mechanistic advances are grounded in the rapidly expanding field of exercise oncology, which systematically investigates the influence of exercise on cancer risk, treatment tolerance, tumor biology, survivorship, and mortality [5]. In this field, systematic reviews and meta-analyses have revealed that exercise has positive effects on physical and mental symptoms, quality of life, and recurrence and survival in patients with cancer [6–10]. These findings have positioned exercise as a potential supportive strategy, alongside standard oncologic treatment [2, 11]. While the mechanisms underlying these effects are not fully understood, previous studies have demonstrated the potential of exercise to regulate intratumoral vascular maturity, perfusion, hypoxia, metabolism, and the anti-tumor immune response [12–14]. Recently, advances in the field of exercise oncology have emphasized the importance of exercise-induced secreted factors, termed "exerkines," such as myokines and adipokines (e.g., interleukin [IL]-6, IL-15, and secreted protein acidic and rich in cysteine [SPARC]). These factors have been proposed to influence tumor biology, either directly or indirectly, by enhancing treatment tolerance through modulating inflammation, metabolism, and tissue cross‑talk [4]. Furthermore, exercise may mitigate treatment-related toxicities, such as cytopenia and peripheral neuropathy, thereby improving treatment tolerance and facilitating the maintenance of key chemotherapy parameters, such as RDI and adherence to the planned dosing schedule, both of which are essential for ensuring treatment efficacy [15].

Despite growing interest in this area, evidence on the effects of exercise on treatment outcomes during cancer treatment remains scarce. Bland et al. [16] conducted a systematic review on the impact of exercise on chemotherapy completion rates in women with early-stage breast cancer. Among the eight studies included, only two reported significant improvements [17, 18], while the remaining six found no such effect [19–24]. In contrast, Yang et al. [25] reported in their systematic review that exercise may improve the objective tumor response, as measured by the Response Evaluation Criteria in Solid Tumors (RECIST) to neoadjuvant chemoradiation in patients with rectal cancer [26]. However, similar benefits were not observed in patients with lymphoma [19] or in those receiving neoadjuvant chemotherapy for breast cancer [27]. These findings suggest that the impact of exercise may vary depending on the cancer type, treatment modality, and outcome measure. Although these previous reviews are informative, they only addressed a limited number of outcomes, i.e., chemotherapy completion rates [16] and RECIST response [25]. Moreover, these reviews did not comprehensively map how treatment efficacy outcomes have been defined and assessed across studies. This is of critical importance because studies examining exercise during cancer treatment have employed diverse exercise interventions, including aerobic exercise alone or in combination with resistance training, delivered in supervised or unsupervised settings, with substantial variations in intensity, frequency, and duration. A comprehensive scoping review with a clearly defined search strategy is warranted to address the broad and heterogeneous body of evidence regarding the effects of exercise.

Therefore, in this scoping review, we aimed to identify, summarize, and map the existing literature on the effects of exercise on treatment efficacy, including RDI, treatment completion, and tumor response, for patients with cancer to highlight areas for future research. Specifically, we addressed the following questions:

i) Which cancer types have been investigated in relation to the effects of exercise on treatment efficacy?
ii) Which types and phases of treatment have been studied regarding the impact of exercise on treatment efficacy?
iii) What types of exercise interventions have been examined in studies assessing their effects on treatment efficacy?
iv) How have treatment efficacy outcomes been defined and measured in studies examining the effects of exercise during cancer treatment?

## Methods

This scoping review was conducted in accordance with the Joanna Briggs Institute scoping review methodology [28]. Reporting adhered to the Preferred Reporting Items for Systematic Reviews and Meta-Analyses Extension for Scoping Reviews (PRISMA-ScR) [29]. The protocol is registered in the Open Science Framework (https://osf.io/24agn/).

### Search strategy

A comprehensive literature search was conducted in accordance with the PRISMA-ScR guidelines by an experienced research librarian. Published literature from database inception to November 6, 2025, was searched using PubMed/MEDLINE, the Cochrane Library, and Cumulative Index to Nursing and Allied Health Literature (CINAHL) Complete. The search strategy focused on three core domains: cancer, exercise, and treatment-related outcomes. A combination of controlled vocabulary terms and free-text keywords, tailored to each database, was used. Full, database-specific search strategies are provided in Additional File 1. The reference lists of the included studies and relevant reviews were screened to identify additional eligible articles.

### Study selection and eligibility criteria

Study selection followed a structured process that included managing search results, removing duplicates, screening titles and abstracts, and performing full-text reviews. We included (1) randomized controlled trials (RCTs), quasi-experimental studies, and observational studies; (2) original human studies; (3) studies examining patients with cancer; (4) studies examining patients treated with chemotherapy, immunotherapy, or radiation therapy; (5) studies examining the effects of exercise on treatment efficacy as outcomes; and (6) studies published in English. Case series, case reports, conference abstracts, review articles, and editorials were excluded because these sources typically lack sufficient methodological detail and quantitative outcome data. Review articles were evaluated, and relevant studies were extracted. After removing duplicates, eight reviewers (TF, JN, KS, KO, TT, TO, JI, and SM) evaluated the eligibility of studies by independently screening the titles and abstracts of potential citations according to the eligibility criteria. The abstracts and titles of full-text articles were screened to assess eligibility or identify studies lacking sufficient information for inclusion. The final inclusion of eligible studies was determined by consensus among all authors.

### Data charting process

Data were charted using a standardized extraction form developed in line with PRISMA-ScR recommendations. Two reviewers (TF and JN) independently charted data from each study using the finalized form. The following variables were extracted: 1) first author’s last name, 2) publication year, 3) nationality, 4) study design, 5) number of patients, 6) age, 7) sex, 8) cancer type, 9) cancer stage, 10) treatment and intervention timing, 11) intervention, 12) exercise adherence, 13) exercise-related adverse events, 14) outcomes of treatment efficacy, and 15) conclusions. Discrepancies between reviewers were discussed and resolved through consensus; if disagreement persisted, a third reviewer was consulted. The extracted data were organized and managed using Microsoft Excel (Microsoft Corp., Redmond, WA, USA).

### Outcomes

Consistent with the objectives of this scoping review, no hierarchy of outcomes (e.g., primary or secondary) was applied. Instead, treatment-related outcomes were identified, classified, and mapped according to how they were defined and assessed in the included studies. For descriptive purposes, the outcomes were organized into two conceptual domains:

1. Treatment delivery and tolerance outcomes, including RDI, dose delays or reductions, and treatment completion
2. Tumor response outcomes, including pathological complete response, RECIST-based response, tumor size, and downstaging

### Synthesis of results

The extracted data are summarized descriptively in tables and figures. The primary objective of this scoping review was to map existing evidence on exercise interventions and treatment-related outcomes, encompassing various cancer types, treatment modalities, intervention characteristics, and outcome measures, rather than estimating pooled effect sizes. Therefore, a meta-analysis was not included in the review protocol because substantial clinical and methodological heterogeneity was anticipated across studies. Data were synthesized descriptively and narratively to capture the breadth and heterogeneity of the available evidence. Studies were compared based on the characteristics of exercise interventions, including supervision, the type of exercise (aerobic, resistance, or combined), exercise intensity, the frequency of sessions, and the duration of the intervention. The synthesis focused on mapping how these characteristics influenced therapeutic outcomes and identifying features of exercise prescriptions associated with treatment-related outcomes. Key findings were summarized both narratively and visually to highlight patterns, variations, and research gaps across the included studies.

## Results

**Fig. 1:**
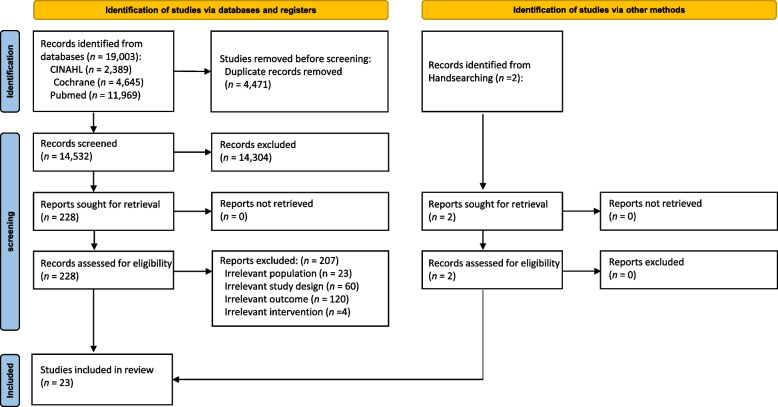
Flow diagram. The diagram depicts the identification, screening, eligibility assessment, and inclusion of studies in accordance with the PRISMA-ScR guidelines

The initial literature search yielded 19,003 articles, of which 14,532 remained after duplicates were excluded. After screening the titles and abstracts, 228 articles were identified as eligible for a full-text review. After full-text review, 207 articles were excluded owing to irrelevant study populations (studies not involving patients with cancer), study design (review articles, case reports, or protocols, or studies examining only the relationship between physical function and treatment outcomes), or outcomes (studies not evaluating treatment efficacy). Additionally, two articles that met the eligibility criteria were identified through a manual search. Consequently, 23 studies were included in this scoping review (Fig. 1).

### Study overview

Table 1 summarizes the characteristics of the included studies. All included articles were published after 2007, and the number of papers has been increasing since the late 2010s. Five studies each were conducted in the United States [27, 30–33] and the Netherlands [18, 21, 22, 34, 35]; four studies were conducted in Canada [17, 19, 24, 26]; two studies each in China [20, 36], South Korea [37, 38], and Denmark [39, 40]; and one study each in Sweden [41], the United Kingdom [42], and Germany [43].

**Table 1:** Characteristics of the included studies

Year Author Country	Study design	Number of patients	Age, mean (SD)	Sex (female)	Cancer type	Stage	Treatment and intervention timing	Intervention: 1) supervision 2) type 3) intensity 4) frequency 5) duration	Exercise adherence	Exercise-related adverse events	Outcomes of treatment efficacy	Conclusion (Main results)
2024 Caan BJ USA [30]	RCT	Total: 181 Ex: 90 Con: 91	Total: 55.2 ± 12.8 Ex: 56.3 ± 12.9 Con: 54.2 ± 12.6	47.5%	Colon	II or III	During adjuvant chemotherapy	1) Unsupervised (Home-based) 2) Resistance exercise six to 10 repetitions for three to five sets (chest press, bent one arm row, squats, lunges, and deadlifts) 3) Moderate-to-high intensity (65–85% of the 1RM) 4) Twice per week 5) During chemotherapy	1.4 (0.6–1.7) sessions per week, 62% (53–70) of the estimated 1RM, three sets (2.6–3.6) with 7.5 repetitions (6.4–8.7)	No serious adverse events	RDI	There were no differences in the mean RDI among those in resistance exercise (79% [SD, 19%]) and those in usual care (82% [SD, 19%]); (mean difference −0.04 [95% confidence interval (CI), −0.09 to 0.02]). Among patients with colon cancer, these results do not support home-based resistance exercise as an adjunct to chemotherapy specifically to improve planned treatment intensity
2023 Potiaumpai M USA [31]	Prospective single arm trial	Total: 105	Total: 58.1 ± 11.6	69.5%	Breast Gastrointestinal Pancreatic	I—IV	During chemotherapy	1) Unsupervised (Home-based) 2) Resistance exercise Aerobic exercise Patients’ exercise prescription was personalized based on different factors including baseline functionality and pre-existing comorbidities and symptomology 3) Not reported 4) Twice per week 5) During chemotherapy	Exercise adherence ≥ 70%: 34.3%	Not reported	RDI Dose reduction Dose delay	Patients with breast cancer had a significantly higher average RDI (89.8% ± 17.6%) compared with gastrointestinal cancer (76.8% ± 20.9%, *p* < 0.01) and pancreatic cancer (65.2% ± 20.1%, *p* < 0.01). Only 25% of breast cancer patents required a dose reduction compared to 56.3% of gastrointestinal cancer and 86.4% of pancreatic cancer patients. Cancer site was significantly associated with RDI. Compared with breast cancer, patients with gastrointestinal cancer (β = − 0.12, *p* = 0.03) and pancreatic cancer (β = − 0.22, *p* < 0.01) achieved significantly lower RDI. Every 2.72 unit increase in overall exercise adherence led to a significant 7% decrease in RDI (*p* < 0.01) in gastrointestinal cancer patients. Metastatic gastrointestinal cancer patients had a 15% RDI increase for every 2.72 unit increase in exercise adherence (*p* = 0.04)
2023 Sanft T USA [32]	RCT	Total: 173 Ex: 87 Con: 86	Ex: 52.3 ± 11.3 Con: 53.3 ± 10.9	100.0%	Breast	I—III	During chemotherapy	1) Unsupervised (Home-based) 2) Resistance exercise Aerobic exercise 3) Moderate- to vigorous-intensity 4) Twice weekly 5) During chemotherapy	Meeting physical activity level: 52%	Not reported	RDI Dose reduction Dose delay Toxicity dose delay pCR	Participants randomly assigned to intervention had greater improvements in exercise and diet quality compared with Con (*p* < 0.05). RDI was 92.9% ± 12.1% and 93.6% ± 11.1% for intervention and Con, respectively (*p* = 0.69); the proportion of patients in the intervention versus Con who achieved ≥ 85% RDI was 81% and 85%, respectively (*p* = 0.44). The proportion of patients who had at least one dose reduction and/or delay was 38% intervention and 36% Con (*p* = 0.80). Among 72 women who received neoadjuvant chemotherapy, women randomly assigned to intervention were more likely to have a pCR than those randomly assigned to Con (53% v 28%; *p* = 0.04)
2022 Zylstra J UK [42]	Prospective non-RCT	Total: 42 Ex: 22 Con: 20	Ex: 64.0 (40–76.3) Con: 65.8 (42.5–77.9)	85.7%	Esophagus	T1-4, N0-3	During neoadjuvant chemotherapy	1) Unsupervised (Home-based) 2) Resistance exercise and aerobic exercise 3) Moderate intensity 4) Not reported 5) During chemotherapy	Not reported	Not reported	Mandard Tumour Regression Grade Downstaging (cTNM-ypTNM)	Comparison of the Intervention and Control groups indicated the Intervention group had higher rates of tumour regression (Mandard TRG 1–3 Intervention *n* = 15/20 (75%) vs Control *n* = 7/19 (36.8%) *p* = 0.03) including adjusted analyses (OR 6.57; 95% CI 1.52 to 28.30). Combined tumour and node downstaging (Intervention *n* = 9 (42.9%) vs Control *n* = 3 (15.8%) *p* = 0.09) were improved
2021 Morielli AR Canada [26]	RCT	Total: 36 Ex: 18 Con: 18	Total: 57 ± 12 Ex: 56 ± 14 Con: 58 ± 9	33%	Rectus	II—IV	During and after neoadjuvant chemoradiationtherapy	1) Supervised 2) HIIT 2-min, high-intensity intervals completed at 85% of VO_2_ peak interspersed with 2 min of active recovery completed at 40% of VO_2_ peak on a treadmill The number of HIIT intervals started with 5 and progressed by 1 every second session until participants reached 8 intervals Total duration of the exercise sessions progressed from 28 to 40 min 3) High intensity 4) 3 times per week 5) During chemoradiationtherapy	Exercise attendance rate: 82% (65–95%)	No serious adverse events	Treatment response (surgical pathology report) Treatment completion	There were no significant differences between groups for grade 3/4 toxicities or treatment completion. Of 18 patients in the exercise group, 10 (56%) achieved pCR/near pCR compared with 3 of 17 (18%) in the usual care group (*p* = 0.02)
2020 Mijwel S Sweden [41]	RCT	Total: 206 Ex1 (resistance): 74 Ex2 (aerobic): 72 Con: 60	Ex1 (resistance): 52.7 ± 10.3 Ex2 (aerobic): 54.4 ± 10.3 Con: 52.6 ± 10.2	100.0%	Breast	I—III	During adjuvant chemotherapy	1) Supervised 2) Ex1: RT-HIIT resistance exercise: cycle ergometer: 3-min bouts of high-intensity interval exercise Ex2: AT-HIIT Aerobic exercise: 20 min, moderate intensity cycle ergometer: 3-min bouts of high-intensity interval exercise 3) Ex1: high intensity (70–80% of 1RM) Ex2: high intensity 4) Twice per week 5) 16 weeks	Exercise attendance rate: Ex1: 68% Ex2: 63% Adherence to intensity: Ex1: 83% Ex2: 75%	Not reported	RDI	No significant between-groups differences were found in the proportion of participants who required dose reductions (RT-HIIT vs. Con: OR, 1.08; AT-HIIT vs. Con: OR, 1.39), or average relative dose intensity of chemotherapy between groups (RT-HIIT vs. Con: ES, 0.08; AT-HIIT vs. Con: ES, −0.07)
2019 Shim YJ South Korea [37]	Prospective controlled study	Total: 39 Ex: 25 Con: 14	Total: < 65: 26 (67.0%) ≥ 65: 13 (33.0%) Ex: < 65: 19 (49.0%) ≥ 65: 6 (15.0%) Con: < 65: 7 (18.0%) ≥ 65: 7 (18.0%)	54.0%	Colon	I—III	During adjuvant therapy (chemotherapy or chemoradiationtherapy)	1) Unsupervised (Home-based) 2) Resistance exercise stand on one leg, lunge, arm curl, squat, lateral raise, and leg raise Aerobic exercise walking, march step, V-step, step touch, skip step, and kick step Two sets of total 12 resistance and aerobic moves were implemented with each move done for a minute and with breaks of 5 or 6 min in between 3) Low intensity (not allowed to exceed 11–14 points by the Borg rating of perceived exertion scale) 4) Three times a week 5) During chemotherapy or chemoradiationtherapy	Not reported	Not reported	Treatment completion (dose reductions or premature termination)	Compared with the control group, a significantly lower proportion of the exercise group failed to complete the prescribed regimen (57% and 20%, respectively, *p* = 0.02). A fivefold lower possibility of dose adjustment in the exercise group compared to the control group was demonstrated (OR, 0.188; *p* = 0.02; 95% CI, 0.044–0.793)
2007 Courneya KS Canada [17]	RCT	Total: 242 Ex1 (resistance): 82 Ex2 (aerobic): 78 Con: 82	Total: 49.2 (range: 25–78) Ex1 (resistance): 49.5 (range: 25–76) Ex2 (aerobic): 49.0 (range: 30–75) Con: 49.0 (range: 26–78)	100.0%	Breast	I—III	During adjuvant therapy (chemotherapy or chemoradiationtherapy)	1) Supervised 2) Ex1: Resistance Two sets of eight to 12 repetitions of nine different exercises (leg extension, leg curl, leg press, calf raises, chest press, seated row, triceps extension, biceps curls, and modified curl-ups) Resistance was increased by 10% when participants completed more than 12 repetitions Ex2: Aerobic Cycle ergometer, treadmill, or elliptical Exercise duration began at 15 min for weeks 1 to 3 and increased by 5 min every 3 weeks until the duration reached 45 min at week 3) Ex1: Moderate-to-high intensity (60% to 70% of 1RM) Ex2: Moderate-to-high intensity (beginning at 60% of their maximal oxygen consumption, or VO_2_ max, for weeks 1 to 6 and progressing to 70% during weeks 7 to 12 and 80% beyond week 12) 4) Three times per week 5) During chemotherapy or chemoradiationtherapy	Attendance rate: Ex1: 72.0% Ex2: 68.2%	Two patients experienced an adverse event related to exercise (after baseline maximal treadmill testing) lightheaded, hypotensive, and moderately nauseous dizziness, weakness, and mild diarrhea	RDI	RDI was 84.1% in the Con group compared with 89.8% in the RET group (mean difference = 5.7%; 95% CI, 0.4% to 11.0%; *p* = 0.03) and 87.4% in the AET group (mean difference = 3.3%;95% CI, −2.5% to 9.2%; *p* = 0.27)
2019 Liu S China [36]	Retrospective	Total: 219 Ex: 116 Con: 103	Total: 63.5 ± 6.4 Ex: ≤ 63: 69 (59.48%) > 63: 47 (40.52%) Con: ≤ 63: 58 (56.31%) > 63: 45 (43.69%)	37.5%	Cholangiocarcinoma	NR	After surgery, adjuvant chemotherapy	1) Not reported 2) Mobilization after surgery, walking, Tai Chi 3) Not reported 4) Not reported 5) Not reported	Not reported	Not reported	RECIST (after 1 month of treatment)	Ex: 10 cases with complete remission, 79 cases with partial remission, 17 cases with stable disease, and 10 cases with progressive disease. The response rate was 76.72%. Con: 3 cases with complete remission, 45 cases with partial remission, 40 cases with stable disease, and 15 cases with progressive disease. The response rate was 46.60%. The response rate in the Ex was significantly higher than that in the Con group (*p* < 0.05)
2022 Sturgeon KM USA [33]	RCT	Total: 19 Ex: 9 Con: 10	Total: 49.4 ± 10.5 Ex: 47.0 ± 11.7 Con: 51.5 ± 9.5	100.0%	Breast	I—III	During neoadjuvant chemotherapy	1) Unsupervised (Home-based) 2) Aerobic exercise 3) Moderate-to-high intensity (Weeks 1–4: 50% of VO_2_max, 60–75 min/wk, Weeks 5–11: 60–80% of VO2max, 60–75 min/wk, Weeks 12–24: maintain the exercise prescription from week 11) 4) Three times per week 5) 16–24 weeks	Adherence: 87.6%	There were no differences in adverse events between the groups	Treatment delay Pathology reports after surgery	There were no significant differences in treatment delays (con: 55%; Ex: 37%), or pathological complete response (con: 67%; Ex: 75%) between groups
2013 Courneya KS Canada [24]	RCT	Total: 301 Ex1 (standard aerobic): 96 Ex2 (high-volume aerobic): 101 Ex3 (aerobic + resistance): 104	Total: 50.0 ± 8.9 Ex1 (standard aerobic): 49.2 ± 8.4 Ex2 (high-volume aerobic): 50.1 ± 8.8 Ex3 (aerobic + resistance): 50.5 ± 9.4	100.0%	Breast	I—III	During adjuvant chemotherapy	1) Supervised 2) Ex1: Standard aerobic 75 min per week cycle ergometer, treadmill, elliptical, rowing ergometer, or combination 25–30 min per session Ex2: High-volume aerobic, 150 min per week Ex3: 3) Ex1: High intensity (55%−70% VO_2_ peak) Ex2: Vigorous intensity Ex3: High intensity (60% to 75% of 1RM) Aerobic + resistance Aerobic: same as standard aerobic Resistance: two sets of 10 to 12 repetitions of nine different strength exercises (leg extension, leg curl, leg press, calf raise, chest press, seated row, triceps extension, biceps curl, and modified curl-up) 50–60 min per session 4) Three days per week 5) During chemotherapy	Aerobic exercise adherence: Ex1: 87.8% Ex2: 81.6% Ex3: 78.0% Resistance exercise adherence: Ex3: 66.0%	No serious adverse events	RDI	RDI was 93.9% in standard aerobic exercise compared with 92.7% in aerobic + resistance and 91.6% in high-volume aerobic (*p* = 0.34)
2022 Mikkelsen MK Denmark [39]	RCT	total: 84 Ex: 41 Con: 43	Ex: 72.1 (IQR: 67.3–74.5) Con: 71.5 (IQR: 68.5–75.3)	57.10%	Pancreas Biliary tract Non-small cell lung	III-IV	During first line chemotherapy	1) Supervised 2) Aerobic and resistance Aerobic Walking program Individualized goal setting and evaluation resistance chest press, abdominal crunch, leg press, leg curl, leg extension, lower back, and low row 2 sets (session 1–6), 3 setrs (session 7–24) 3) moderate intensity (15 RM: session 1–2, 12 RM: session 3–13, 10 RM: session 14–24) 4) Two times per week 5) 12 weeks	Adherence: Resistance exercise 69% (IQR 21%−88%) Aerobic exercise 75% (IQR 33%−100%)	Grade 1: 5 (bruises and feeling sick/dizzy) Grade 2: 1 (swollen knee) Grade 3: 2 (osteoporotic spinal compression and spinal stenosis)	RDI	No significant differences were seen for dose intensity of any chemotherapy regimens
2009 Courneya KS Canada [19]	RCT	total: 122 Ex: 60 Con: 62	Ex: 52.8 (range: 18–77) Con: 53.5 (range: 18–80)	41.00%	Lymphoma	I-IV	During chemotherapy or no treatments	1) Supervised 2) Aerobic exercise Cycle ergometer 3) Moderate-to-high intensity (60% of the peak power output (increased by 5% each week to 75% by the fourth week) 15 to 20 min for the first 4 weeks (increased by 5 min per week to 40 to 45 min in the ninth week) 4) Three times per week 5) 12 weeks	Attendance rate: Mean: 77.8% (28.0 of 36) Median: 91.7% (33.0 of 36)	No serious events but three adverse events (back, hip, and knee pain) were related to exercise	Chemotherapy completioin rate	For participants receiving chemotherapy (*n* = 54), the Ex group (*n* = 28) completed 103% of its planned minimum and 94% of its planned maximum cycles compared with 99% (*p* = 0.45) and 89% (*p* = 0.20) for Con (*n* = 26), respectively
2012 Rao R USA [27]	RCT	total: 10 Ex: 5 Con: 5	Ex: 59.8 Con: 51.4	100.0%	Breast	II—III	During neoadjuvant chemotherapy	1) Supervised 2) Resistance and aerobic exercise Jumping jacks, running in place, arm and leg work with exercise balls, bands, and weights up to 5 pounds 48 sessions 3) Not reported 4) Three times per week 5) 16 weeks	All five patients in the bootcamp group completed > 80% of the advised exercise sessions	Not reported	Tumor size	No significant differences were seen for tumor size
2015 van Waart H Netherlands [18]	RCT	Total: 230 Ex1 (low-intensity): 77 Ex2 (moderate- to high-intensity): 76 Con: 77	Total: 50.7 ± 9.1 Ex1 (low-intensity): 50.5 ± 10.1 Ex2 (moderate- to high-intensity): 49.9 ± 8.4 Con: 51.6 ± 8.8	99.0%	Breast	I—III	During adjuvant chemotherapy	1) Ex1: Unsupervised (Home-based) Ex2: Supervised 2) Ex1: Aerobic exercise physical activity program (30 min/day) Ex2: Resistance and aerobic exercise Six large muscle groups were trained for 20 min per session, with two series of eight repetitions, and 30 min of aerobic exercises 3) Ex1: Low-intensity Ex2: Moderate- to high-intensity (Resistance: 80% of the 1RM, Aerobic: intensity of 50% to 80% of the maximal workload as estimated by the Steep Ramp Test) 4) Ex1: 5 days/week Ex2: 2 sessions per week 5) During chemotherapy and continued until 3 weeks after the last cycle	Attendance rate: Ex2: 71% Adherence of the recommendations regarding daily activity levels at least 75% of the time: Ex1: 55% Ex2: 48%	Not reported	Chemotherapy completion rates, dose reduction	A significantly smaller percentage of moderate- to high-intensity exercise (12%) required dose adjustments in the prescribed chemotherapy regimen than Con (34%) or low-intensity exercise (34%; OR, 0.26; *p* < 0.01), indicating about a fourfold lower likelihood of dose adjustment; 95% CI, 0.11 to 0.61 for both comparisons). The average dose reduction among those who required chemotherapy adjustment in moderate- to high-intensity exercise and low-intensity exercise was 10%, compared with 25% in Con (mean difference, 0.15; 95% CI, 2.96 to 0.01; *p* = 0.01)
2018 van Waart H Netherlands [22]	RCT	Total: 23 Ex1 (low-intensity): 8 Ex2 (moderate- to high-intensity): 7 Con: 8	Total: 58.2 ± 10.1 Ex1 (low-intensity): 60.1 ± 7.3 Ex2 (moderate- to high-intensity): 57.7 ± 13.2 Con: 56.7 ± 10.6	61.0%	Colon	II—IV	During adjuvant chemotherapy	1) Ex1: Unsupervised (Home-based) Ex2: Supervised 2) Ex1: Aerobic exercise physical activity program (30 min/day) Ex2: Resistance and aerobic exercise Six large muscle groups were trained for 20 min per session, with two series of eight repetitions, and 30 min of aerobic exercises 3) Ex1: Low-intensity Ex2: Moderate- to high-intensity (Resistance: 80% of the 1RM, Aerobic: intensity of 50% to 80% of the maximal workload as estimated by the Steep Ramp Test) 4) Ex1: 5 days/week Ex2: 2 sessions per week 5) During chemotherapy	Attendance rate: Ex2: 61% Adherence of the recommendations regarding daily activity levels at least 75% of the time: Ex1: 100% Ex2: 71%	No adverse events	Chemotherapy completion rates	Participants in moderate- to high-intensity exercise received, on average, 87% (SD 15%), in low-intensity exercise, 92% (SD 8%), and in Con, 78% (SD 16%) of the planned dose
2015 Xu YJ China [20]	RCT	Total: 56 Ex: 28 Con: 28	Total: Ex: 58.1 ± 9.6 Con: 61.1 ± 9.0	7.10%	Esophagus	I—III	During neoadjuvant chemoradiotherapy	1) Supervised 2) Aerobic exercise Walking program (20 min) 3) Low-intensity (60% of maximal HR) 4) Three times per week 5) During chemoradiotherapy	Adherence rate: 68% (range: 32%–100%)	Not reported	Chemotherapy completion rates	No significant differences were seen for chemotherapy interruption
2016 Van Vulpen JK Netherlands [21]	RCT	Total: 33 Ex: 17 Con: 16	Total: Ex: 58.1 ± 10.3 Con: 58.1 ± 9.6	36.40%	Colon	M 0	During adjuvant chemotherapy	1) Supervised 2) Aerobic and Resistance 60 min Aerobic: interval training of alternating intensity Resistance exercise: arms, legs, shoulders, and trunk 3) Moderate-to-high intensity (Aerobic: HR at (3 × 2 min increasing to 2 × 7 min) and below (3 × 4 min decreasing to 1 × 7 min) ventilatory threshold. Resistance: 65–75% of 1RM) 4) Two times per week 5) 18 weeks	Adherence rate: 89% (IQR, 72%–97%)	No serious adverse events	RDI	RDI was 82% (IQR, 74–87) in the intervention group, compared with 76% (IQR, 65–91) in the usual care group (*p* = 0.80)
2025 Brouwer CG Netherlands [34]	RCT	Ex: 39 Con: 39	Ex: 55.4 ± 10.5 Con: 59.7 ± 11.0	100%	Ovarian	I—IV	During neoadjuvant or adjuvant chemotherapy	1) Supervised 2) Aerobic and Resistance 60 min Aerobic: 30 min cycle ergometer Resistance exercise: vertical row, leg press, bench press, pull over, abdominal crunch, and lunge 3) Moderate-to-high intensity (Aerobic: starting at an intensity of 50% with a maximum of 80% of the maximal workload. Resistance: two sets of 10 repetitions, at 70–80% of the maximum workload estimated with the indirect 1RM) 4) Two times per week 5) During chemotherapy (about 18 weeks)	Attendance rate: median of 72% (52–85)	Not reported	RDI (dose reduction, dose delay)	The proportions of patients with ovarian cancer with a relative dose intensity ≥ 85% and an 18-month progression-free survival were numerically higher in the intervention group compared with the control group, but these differences were not statistically significant
2025 Mast IH Netherlands [35]	RCT	EX1: 10 EX2: 10 Con: 11	EX1: 68 ± 7 EX2: 62 ± 9 Con: 66 ± 7	16%	Esophageal	cT: 2–3 cN: 0–3	During neoadjuvant chemoradiotherapy	Ex1: 1) Supervised 2) Aerobic Aerobic: 30 min cycle ergometer 3) Moderate intensity 50%−70% of the estimated Wmax, adjusted to Borg 12–14 4) Five times per week 5) 1 h prior to the scheduled radiotherapy (about 5 weeks) Ex2: 1) Supervised 2) Aerobic and Resistance: 50 min Aerobic: 30 min cycle ergometer Resistance exercise: 6 large muscle groups 3) Moderate-to-high intensity Aerobic: 50% 80% of estimated Wmax, adjusted to Borg 12–15 if needed Resistance: 2 sets of 8 12 repetitions at 70% 80% of their estimated 1RM 4) Two times per week (Patients were asked to perform additional one moderate intensity aerobic exercise at home) 5) During chemoradiotherapy	Attendance rate: Ex1: 98% (IQR 96–100%) Ex2: 78% (IQR 33–100%)	No serious adverse events	Full dose, tumor response	A good tumor response was observed in 70% of patients in the Ex1 and 2 compared with 55% in the Con (odds ratio [OR] = 1.9, 85% CI: 0.5–7.7)
2025 Hong MJ Korea [38]	RCT	Ex: 14 Con: 12	Ex: 69.46 (63.5–74.5) Con: 64.68 (58.0–73.0)	11.5%	Lung	III—IV	During chemotherapy	1) Unsupervised 2) Aerobic and Resistance (plus stretching and respiratory training) 60 min Aerobic: walking or running Resistance exercise: squats, bridge exercises, and upper limb strength training with bands (targeting shoulder flexors, extensors, abductors, and elbow movements) 3) Moderate-to-high intensity (Aerobic: 50%–60% of maximal heart rate. Resistance: training program with weights was recommended based on individual assessments) 4) Three times per week 5) During chemotherapy	Average frequency sessions: 3.13 times per week	Not reported	RECIST, dose reduction or delay	Delays in chemotherapy occurred in 40.0% of participants in the control group but none in the exercise group (*p* = 0.02). Additionally, two cases of chemotherapy discontinuation and one of dose reduction occurred in the control group
2025 Schmidt ME Germany [43]	RCT	EX1: 60 EX2: 60 Con: 60	EX1: 48.4 ± 10.9 EX2: 51.4 ± 11.2 Con: 49.7 ± 9.9	100%	Breast	cN: 0–3	During neoadjuvant chemotherapy	EX1: 1) Supervised 2) Resistance exercise: leg extension, leg curl, leg press, shoulder internal and external rotation, seated row, latissimus pull down, butterfly, and butterfly reverse 3) Moderate-to-high intensity (3 sets of 8 12 repetitions with a weight that could be lifted for 8‒12 repetitions, corresponding to 60% 80% of the participant’s 1RM) 4) Two times per week (Patients were instructed to perform additional one weekly resistance home-exercise) 5) During chemotherapy EX2: 1) Supervised 2) Aerobic Aerobic: cycle ergometer 3) Moderate-to-high intensity (Weeks 1 and 2: Each session lasted 15–30 min. The intensity was set at 60% of the patient’s VO2peak. Weeks 3–6: Session duration increased to between 30 and 60 min. The intensity gradually increased, reaching 70% of VO2peak by Week 6. Week 7 onwards: Each session lasted approximately 30 min and utilized interval training consisting of 4 higher intensity intervals at 75%- 85% VO2peak for 4 min, interspersed with 3-min recovery intervals at 60% VO2peak.) 4) Two times per week (Patients were instructed to perform additional one weekly aerobic home-exercise) 5) During chemotherapy	Attendance above 75% of scheduled sessions twice weekly over the whole course of neoadjuvant chemotherapy was reached by only 12% in EX1 and 13% in EX2	Not reported	Tumor size, pCR, and RDI	Overall, there was no significant difference in post-intervention tumor size between EX1 or EX2 and Con. However, there was a significant effect modification by hormone receptor (HR) status (p interaction = 0.03). Among patients with HR + tumors, results suggest a beneficial effect of EX2 on tumor shrinkage (odds ratio (OR) = 2.37, 95% confidence interval (95%CI): 0.97‒5.78), and on pCR (OR = 3.21, 95%CI: 0.97‒10.61) compared to Con. The effects of EX1 were slightly less pronounced. For HR − subtypes, beneficial effects on RDI were found for EX2 (OR = 3.71, 95%CI: 1.20‒. Both EX2 and EX1 had favorable effects on premature discontinuation of chemotherapy (OR (no vs. yes) = 2.34, 95%CI: 1.10‒5.06), irrespective of tumor receptor status
2025 Kjeldsted E Denmark [40]	RCT	Ex: 50 Con: 52	Ex: 50.5 (42.0–57.7) Con: 52.5 (45.0–61.0)	100%	Breast	Not reported	During neoadjuvant chemotherapy	1) Supervised 2) Aerobic and Resistance Aerobic: HIIT Resistance exercise: pull down, chest press, and leg press 3) Moderate-to-high intensity (Aerobic: a 5 min warm-up followed by four intervals of high intensity cycling lasting 2 min each, separated by 4-min periods of low intensity cycling, and concluded with a 5-min cooldown. Intervals of high intensity were primarily guided by reaching ≥ 85% of the maximum heart rate. Resistance: 12 to 15 repetitions with 65% of 1RM in weeks 1 to 3 followed by an increase in load of 10% from week 4 onward if the participant could complete ≥ 17 repetitions with good quality) 4) Two times per week (encourage to attend all three sessions weekly) 5) During chemotherapy	The adjusted attendance relative to the prescribed two exercise sessions a week was 75% (IQR, 54%–85%), whereas when relative to the encouraged three exercise sessions a week, it was 65% (IQR, 42%–77%)	No serious adverse events	Tumor size, pCR, RDI, dose reduction, and dose delay	No between-group differences in median tumor size change from baseline to presurgery (Ex vs. Con −3.0 mm [95% confidence interval (CI), −8.0 to 14.0]), the proportion with radiologic complete response [Ex 65% vs. Con 56%; odds ratio 1.16 (95% CI, 0.39–3.91)], or pathologic complete response [Ex 59% vs. Con 56%; odds ratio 1.03 (95% CI, 0.43–2.46)]. The exercise program was associated with higher RDI, and fewer dose delays

*Con* Control, *ES* Effect Size, *Ex* Exercise, *HIIT* High-Intensity Interval Training, *HR* Heart rate, *IQR* Interquartile range, *OR* Odds Ratio, *pCR* Pathological Complete Response, *RCT* Randomized Controlled Trial, *RDI* Relative Dose Intensity, *RECIST* Response Evaluation Criteria In Solid Tumors, *RM* Repetition Maximum, *RT* Resistance Training, *SD* Standard Deviation

### Types of cancer

**Fig. 2:**
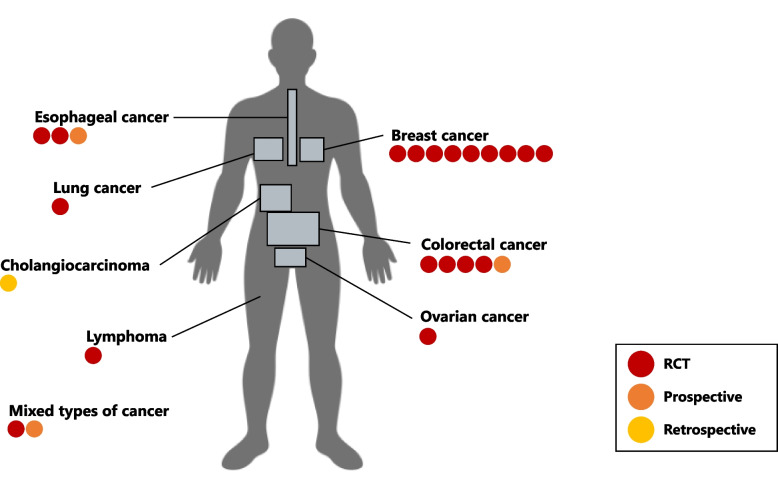
Types of cancer and study design. Visual representation of study designs concerning the effects of exercise on treatment outcomes for each cancer type

Breast cancer was the most frequently studied cancer type, with nine papers (all RCTs) [17, 18, 24, 27, 32, 33, 40, 41, 43]. Colorectal cancer was studied in five papers (four RCTs and one prospective study) [21, 22, 26, 30, 37], and esophageal cancer in three papers (two RCTs and one prospective study) [20, 35, 42]. Cholangiocarcinoma (RCT) [36], lymphoma (retrospective study) [19], ovarian cancer (RCT) [34], and lung cancer (RCT) were investigated in one study each [38]. Mixed types of cancer were studied in two papers (one RCT and one prospective study) [31, 39] (Fig. 2).

### Treatment and intervention phase

All 23 papers included chemotherapy or chemoradiotherapy. Nine studies were conducted in the adjuvant setting [17, 18, 21, 22, 24, 30, 36, 37, 41], eight in the neoadjuvant setting [20, 26, 27, 33, 35, 40, 42, 43], one in the neoadjuvant or adjuvant chemotherapy setting [34], and five during chemotherapy [19, 31, 32, 38, 39]. Although the search strategy included studies on immunotherapy, no trials exclusively evaluating immunotherapy were identified.

### Types of exercise

**Fig. 3:**
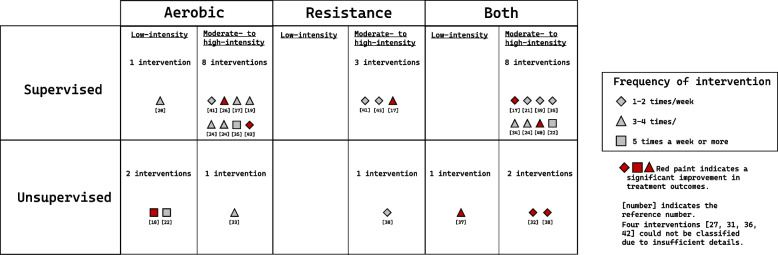
Summary of the effects of exercise on treatment outcomes. Exercise interventions categorized by characteristics, such as supervision, type, intensity, frequency, and treatment efficacy. The association between exercise interventions and treatment-related outcomes in patients with cancer is illustrated in this figure. The figure highlights significant results in red. This figure summarizes the observed patterns across all the research and is descriptive when integrated. These associations are hypothesis-generating and should not be interpreted as evidence of causal relationships

Of the 23 papers, six studies were included in the two-exercise arm [17, 18, 22, 35, 41, 43], one study was included in the three-exercise arm [24], and 31 exercise interventions were pooled. In terms of exercise type, there were 13 aerobic interventions [17–20, 22, 24, 26, 33, 35, 36, 41, 43], four resistance interventions [17, 30, 41, 43], and 14 combinations of aerobic and resistance interventions [18, 21, 22, 24, 27, 31, 32, 34, 35, 37–40, 42]. Twenty-one interventions were supervised [17–22, 24, 26, 27, 34–36, 38–41, 43], nine were unsupervised [18, 22, 30–33, 37, 38, 42], and one was unknown [36]. Concerning the exercise intensity, 24 interventions were moderate- to high-intensity [17–19, 21, 22, 24, 26, 30, 32–35, 38–43], five interventions were low-intensity [18, 20, 22, 31, 37], and two were unknown [27, 36]. The frequency of exercise ranged from one to two times per week for 11 interventions [18, 21, 30–32, 35, 39, 41, 43], three to four times per week for 14 interventions [17, 19, 20, 24, 26, 27, 33, 34, 37, 38, 40], five or more times per week for four interventions [18, 22, 35], and was unknown for two interventions [36, 42]. The duration was specified for seven interventions: two lasted 12 weeks [19, 39], three lasted 16 weeks [27, 41], and one lasted 16–24 [33] or 18 weeks [21]. The remaining 23 interventions were conducted during treatment [17, 18, 20, 22, 24, 26, 30–32, 34, 35, 37, 38, 40, 42, 43], and one intervention was not reported [36] (Table 1, Fig. 3).

### Exercise adherence and safety

Exercise adherence and safety are described in Table 1. Seventeen papers reported exercise adherence [17–22, 24, 26, 33–35, 39–41], with three papers calculating the proportion of participants who met the criteria [27, 31, 43]. Exercise adherence ranged from 61 to 98% [17–22, 24, 26, 33–35, 39–41]. In reports using a 70% adherence rate as the criterion, 34% of cases met the standard [31]. Another study reported that 12–13% met the 75% adherence criterion [43], while 100% met the 80% criterion [27]. Session frequency was reported in two papers [30, 38]. Reports prescribing twice-weekly interventions reported an average of 1.3 completed sessions [30], while those prescribing three weekly interventions reported an average of 3.13 completed sessions [38]. One paper reported adherence to the recommended level of physical activity, with 52% of participants meeting the standard [32]. Exercise adherence was not reported in three papers [36, 37, 42]. Regarding safety, 12 papers did not report adverse events [18, 20, 27, 31, 32, 34, 36–38, 41–43], and nine reported none [21, 22, 24, 26, 30, 33, 35, 40]. Three studies reported adverse events related to exercise, including lightheadedness, hypotension, moderate nausea, dizziness, weakness, and mild diarrhea [17]; osteoporotic spinal compression, and spinal stenosis [39]; and back, hip, and knee pain [19]. Adverse events included hematoma, nausea or dizziness, and swollen knee; however, the original study did not explicitly attribute these events to the exercise intervention [39].

### Types of treatment outcomes

RDI, including dose delay and reduction, was used as an indicator of treatment outcome in 15 papers [17–19, 21, 24, 30–35, 38–41, 43]. Treatment completion was assessed in six studies [18–20, 22, 26, 37]. Pathological complete response was used in seven papers [26, 32, 33, 35, 40, 42, 43]. Tumor size was used in three papers [27, 40, 43], RECIST was used in two papers [36, 38], and downstaging [42] was used in one paper.

## Summary of the effects of exercise on treatment outcomes

We categorized exercise interventions according to their characteristics (supervision, type, intensity, and frequency; Fig. 3). We further summarized the effects by outcome category to clarify which interventions were associated with beneficial treatment outcomes. A beneficial effect was defined as a statistically significant improvement in at least one treatment-related outcome compared with the control group. Overall, nine of the 31 interventions met the criterion, whereas no significant differences between groups were reported for 22 interventions.

### RDI and treatment completion

Of the 28 interventions for which RDI and/or treatment completion were reported, eight demonstrated significant benefits of exercise, while 20 showed no effect. To better contextualize these findings, we compared the characteristics between studies reporting beneficial versus null effects. Moderate-to-high-intensity aerobic exercise (one to two times per week [43] or three to four times per week) [26], resistance training (three to four times per week) [17], and combined exercise (one to two times per week [18] or three to four times per week [40]) under supervision were effective for RDI and/or treatment completion in patients with breast, colorectal, and lung cancer. In an unsupervised setting, moderate-to-high-intensity aerobic and resistance exercise (one to two times per week) [38], low-intensity exercise (three to four times per week) [37], and low-intensity aerobic exercise (five times per week or more) [18] was effective for RDI and/or treatment completion. In contrast, interventions reporting null findings were characterized by heterogeneous exercise prescriptions. No single study characteristic consistently distinguished interventions that reported beneficial effects from those that reported null findings. This highlights substantial clinical and methodological heterogeneity across studies.

### Pathological complete response, RECIST, tumor size, and downstaging

Of the 10 interventions for which pathological complete response, RECIST, tumor size, and/or downstaging were reported, four demonstrated significant benefits of exercise, while six did not. Two types of exercises were effective for improving pathological complete response or tumor size in patients with breast and esophageal cancer: Unsupervised, self-reported moderate-to-high-intensity aerobic and resistance exercise performed once to twice weekly [32], or at an unknown frequency [42], and supervised, objectively measured moderate-to-high-intensity aerobic exercise performed once or twice weekly [43]. Although detailed information on exercise prescription is lacking, one study has shown that aerobic exercise is effective for RECIST in patients with cholangiocarcinoma [36]. In contrast, studies reporting null findings were limited to breast cancer and involved different exercise prescriptions. No single study characteristic distinguished interventions reported to have beneficial effects from those reporting no effects, underscoring significant clinical and methodological heterogeneity among studies.

In summary, favorable treatment outcomes were most frequently reported in studies involving breast and colorectal cancer. Moderate-to-high-intensity exercise was more commonly reported among interventions associated with favorable treatment-related outcomes, with combined aerobic and resistance training prescribed in approximately half the studies. The most common exercise frequency was one to two or three to four times per week. Moreover, the interventions that incorporated both low-intensity aerobic and resistance exercise were performed three to four times per week, while low-intensity aerobic exercise was performed five times per week. The chemotherapy period in the included studies has often been referred to as the intervention period, but specific details remain unknown (Fig. 3). In addition, four studies reporting favorable outcomes associated with exercise interventions provided insufficient detail regarding the exercise prescription [36, 42]. Specifically, a study on supervised aerobic and resistance training conducted three times weekly found no benefit for tumor size; however, the intensity of the exercise was not specified [27]. Similarly, a study examining a twice-weekly, home-based intervention combining aerobic and resistance training for various types of cancer did not clearly specify the intensity of the exercises [31]. Owing to the limited evidence on associations between exercise interventions and treatment-related outcomes among the included studies, definitive conclusions regarding exercise prescription details for specific cancer types could not be drawn within the scope of this scoping review.

### A summary mapping of exercise modality, treatment type, and outcome category for interventions demonstrating beneficial effects

Table 2 presents a summary mapping of exercise modality, treatment type, and outcome category for interventions demonstrating beneficial effects. Nine of the 31 interventions showed significant improvements in at least one treatment-related outcome. Table 2 also shows the combinations of exercise type and intensity that were most frequently associated with favorable outcomes in adjuvant, neoadjuvant, and chemotherapy settings. One intervention was excluded from Fig. 3 due to insufficient FITT details. However, since it demonstrated a beneficial effect, it is included in Table 2 for completeness. Beneficial effects were observed in treatment delivery and tumor response outcomes across these interventions. Most interventions were conducted during chemotherapy, and most involved a combination of aerobic and resistance exercises. These findings suggest that combined exercise during chemotherapy is most consistently associated with favorable treatment-related outcomes.

**Table 2:**
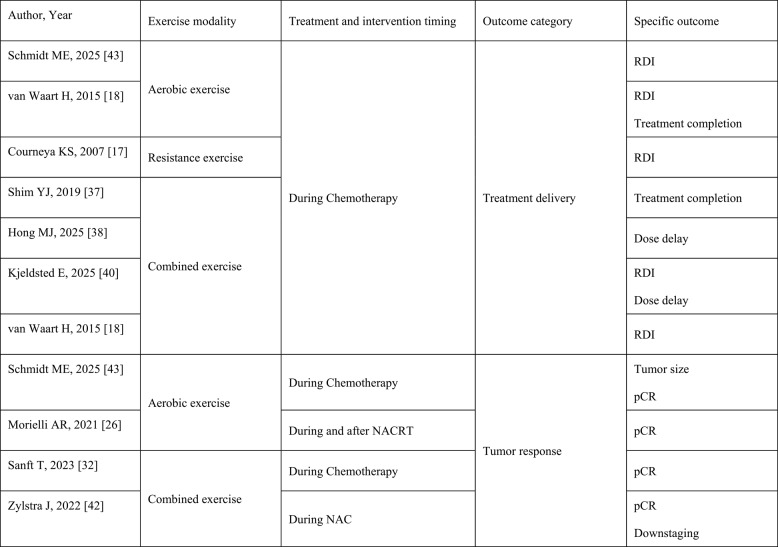
Summary mapping of exercise modality, treatment type, and outcome category for interventions demonstrating beneficial effects

*NAC* Neoadjuvant chemotherapy, *NACRT* Neoadjuvant chemoradiotherapy, *pCR* Pathological Complete Response, *RDI* Relative Dose Intensity

## Discussion

In this scoping review, we mapped the breadth and characteristics of the available evidence examining associations between exercise during chemotherapy or chemoradiotherapy and treatment-related outcomes in patients with cancer. Consistent with the exploratory nature of scoping reviews, we did not estimate pooled effect sizes or formally assess study quality. Given the substantial heterogeneity in cancer types, treatment contexts, exercise prescriptions, and outcome definitions across studies, a quantitative synthesis was not undertaken. Our findings should thus be considered descriptive rather than confirmatory. Accordingly, we avoided causal interpretations and did not perform formal risk-of-bias assessments, which aligns with the exploratory aim of this review.

For conceptual clarity, the treatment-related outcomes discussed in this review can be situated within a broader oncological outcome framework comprising three interrelated domains. First, treatment delivery and tolerance outcomes (e.g., relative dose intensity and treatment completion) reflect the feasibility and continuity of systemic therapy administration. Second, biological response outcomes (e.g., pathological complete response or RECIST-based tumor response) capture tumor-level changes that may arise from maintained or optimized treatment exposure. Third, downstream clinical outcomes (e.g., recurrence or survival) represent the ultimate patient-centered endpoints. However, these outcomes were beyond the scope of this scoping review. This framework is not intended to impose a hierarchy of outcomes but rather to illustrate how exercise-related effects on treatment delivery and tumor response may relate to longer-term clinical outcomes evaluated in other study designs.

Of the 31 interventions, nine demonstrated beneficial effects, primarily in patients with breast and colorectal cancers. Moderate-to-high-intensity exercise, often combining aerobic and resistance training and performed one to two or three to four times per week under supervision, was most frequently associated with improvements in treatment-related outcomes. Unsupervised interventions with lower-intensity or higher-frequency exercise showed benefits in some studies, suggesting that exercise prescriptions may be flexible while supporting treatment outcomes.

These findings indicate that exercise programs incorporating guideline-recommended components, such as moderate-to-high-intensity exercise, combined aerobic and resistance training, and multiple weekly sessions [44], may be associated with treatment delivery and tolerance, potentially through enhanced physical fitness and mitigation of treatment-related toxicities, and may influence tumor response outcomes via systemic immune modulation. Although the optimal intervention duration could not be determined, we observed that moderate-to-high-intensity combined aerobic and resistance training performed multiple times per week may represent an effective approach for improving treatment-related outcomes in patients with cancer. Exercise prescriptions aligned with existing guidelines may remain beneficial when evaluating outcomes related to treatment delivery and tolerance, as well as tumor response. Nevertheless, only individuals capable of performing moderate-to-high-intensity exercise therapy may have been targeted in the related studies. Thus, selection bias cannot be ruled out. In addition, our results do not clarify whether supervision enhances effectiveness. Reported adherence ranged from 61 to 98%, with inconsistent impact on treatment efficacy. Nonetheless, ensuring high adherence is clinically important, and designing programs to maximize adherence may be beneficial. These findings may be viewed through the lens of total training volume and supervision, which provides a unifying framework for understanding the heterogeneity across studies, especially when comparing interventions with different intensity–frequency combinations. Although low-intensity exercise is generally not recommended, two studies have reported its beneficial effects [18, 37]. Notably, van Waart et al. provided only aerobic exercise at a higher-than-recommended frequency [18], suggesting that adjustments in exercise prescription, particularly the overall exercise volume, may influence outcomes. This perspective is consistent with exercise physiology principles, where total training volume is considered an important stimulus for physiological adaptation [45]. However, efficacy was not consistently observed even with similar prescriptions; moreover, a wide range of prescriptions was implemented in reports that did not demonstrate efficacy. Overall, the most effective type of exercise for treatment outcomes remains to be determined, thus warranting further research.

Most of the evidence supporting the efficacy of exercise as a treatment comes from studies of breast and colorectal cancers. The high prevalence of these cancers, their favorable prognoses, and the feasibility of interventions have facilitated research, particularly concerning physical function outcomes [44]. Furthermore, a previous study reported that breast and colorectal cancers are representative types for which exercise lowers the risk of mortality [46]; thus, exercise prescription is indicated for these populations. However, it remains unclear whether these findings can be generalized to other cancer types, including those with poorer prognosis or rarer incidence, warranting further research.

**Fig. 4:**
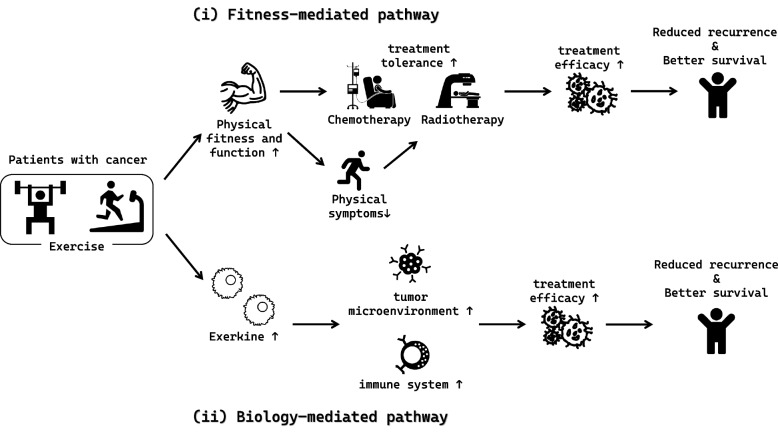
Conceptual pathways through which exercise may influence treatment-related outcomes in patients with cancer. This conceptual model depicts two non-mutually exclusive mechanisms: (i) a fitness-mediated pathway, whereby exercise enhances physical function and reduces symptom burden, potentially improving treatment tolerance and completion; and (ii) a biologically mediated pathway, in which exercise-induced circulating factors (e.g., exerkines) may modulate immune function and the tumor microenvironment, thereby potentially enhancing responsiveness to systemic therapies. This figure is intended to generate and illustrate hypotheses and should not be interpreted as evidence of established causal relationships

A previous study suggested that physical function is associated with prognosis in patients with cancer [47]. In a broader oncological context, previous reviews have reported that higher levels of physical activity are associated with a lower risk of cancer-related mortality [48]. While these findings do not directly address treatment efficacy outcomes examined in the present review, they underscore the potential relevance of exercise intensity and volume in cancer care. Although the mechanisms underlying this relationship are not fully understood, it has been hypothesized that improvements in physical fitness and function, potentially achieved through moderate-to-high-intensity exercise, may increase tolerance to chemotherapy and reduce treatment-related adverse events, including physical symptoms, such as fatigue and dyspnea [6, 16, 49, 50]; this, in turn, may contribute to improved survival [51, 52]. Beyond fitness-mediated effects, preclinical evidence suggests that exercise may modulate biological processes through circulating factors collectively termed "exerkines," including myokines and adipokines. These exerkines have been associated with tumor suppression, immune modulation, and the regulation of cancer-associated cachexia. A recent critical review emphasized the potential roles of exerkines, such as IL-6, IL-15, and SPARC, in mediating direct anti-tumor effects and improving host metabolic and inflammatory status [4]. These improvements may enhance tolerance to systemic cancer therapies. In addition to exerkine-mediated pathways, emerging preclinical immuno-oncology studies suggest that exercise can directly enhance anti-tumor immune responses [4]. Experimental models have demonstrated that exercise can increase the infiltration of CD8⁺ T cells and the activity of natural killer cells within the tumor microenvironment, as well as modulate immune checkpoint signaling. A recent murine study combining aerobic exercise with anti-PD-L1 antibody therapy reported synergistic tumor control, providing evidence that exercise may sensitize tumors to systemic immunotherapy by modulating the immune system [14]. Beyond preclinical observations, clinical studies have begun to explore immune-mediated pathways. A recent systematic review and meta-analysis in patients with breast cancer examined the effects of exercise on tumor-specific immune cells, including natural killer cells and cytotoxic T lymphocytes. Although pooled analyses did not demonstrate statistically significant changes, several of the studies included in that review reported trends toward favorable modulation of immune cell number or activity, and importantly, no detrimental effects [53]. It is important to emphasize that these mechanistic explanations are hypothesis-generating and have not been proven in clinical settings. However, they provide a conceptual framework that links exercise, immune modulation, and observed treatment outcomes. Although they may conceptually align with favorable outcomes observed in certain interventions—such as improved pathological complete response in breast cancer—direct experimental evidence supporting this link is currently lacking. These proposed mechanisms may potentially modulate the tumor microenvironment and enhance responsiveness to systemic therapies, including immune checkpoint inhibitors; however, this remains to be confirmed in clinical studies [54–56]. Based on the findings of this scoping review and existing clinical and preclinical studies, we propose two complementary hypotheses regarding how exercise may influence treatment efficacy in patients with cancer: (i) Exercise that improves physical fitness and function may enhance treatment tolerance, partly through the alleviation of physical symptoms such as fatigue and dyspnea, and thereby contribute to improved clinical outcomes; and (ii) exercise may influence the immune system and tumor microenvironment through exerkine-mediated and immune-related mechanisms, potentially augmenting the efficacy of systemic therapies (Fig. 4). Further research is necessary to clarify the relative contributions of these pathways and to determine how various components of exercise prescription, such as type, intensity, and dosage, affect treatment response in patients with cancer.

### Methodological fragility of the current evidence

Despite growing interest in exercise during cancer treatment, the methodological characteristics of the existing evidence base warrant careful consideration when interpreting the findings of this scoping review. Several features of the primary studies directly affect causal inference and the strength of the observed signals.

First, many included trials were small and likely underpowered to detect modest yet clinically meaningful effects on treatment-related outcomes. In this context, statistically significant findings observed in a subset of interventions—particularly for secondary or exploratory outcomes, such as tumor response—should be interpreted with caution, as they may be unstable and susceptible to chance.

Second, although incomplete reporting and heterogeneity of exercise prescriptions are common limitations in this field, their implications extend beyond generalizability. Intervention components—including intensity, frequency, duration, progression, and adherence—were frequently reported as “unknown.” Such insufficient reporting limits reproducibility, precludes meaningful assessment of dose–response relationships, and complicates interpretation of associations between exercise and treatment-related outcomes. Accordingly, apparent associations between specific exercise characteristics and favorable outcomes cannot be interpreted as evidence of superiority or effectiveness.

Third, the nature of exercise interventions makes participant and provider blinding challenging in most trials, resulting in an inherently elevated risk of performance bias. Moreover, outcomes related to treatment delivery—such as relative dose intensity or treatment completion—may be influenced by clinical decision-making, patient motivation, and contextual factors, thereby complicating attribution of observed effects to the exercise intervention itself.

Fourth, binary classification of interventions as “beneficial” versus “null” oversimplifies the evidence. Defining benefit solely based on statistical significance in at least one outcome does not account for multiplicity, effect size, or clinical relevance and implicitly treats all outcomes as equally important. This approach may overestimate the apparent success of interventions and obscure the consistency and direction of effects.

Finally, as null findings predominated across cancer types, treatment phases, and outcomes, the few statistically significant results may reflect small-study effects, selective outcome reporting, or chance rather than consistent intervention effects.

Taken together, these considerations indicate that the current evidence base is hypothesis-generating rather than confirmatory. Although this review provides a comprehensive mapping of how exercise has been examined in relation to treatment delivery and tumor response outcomes, robust conclusions regarding effectiveness and optimal exercise prescriptions will require adequately powered trials with standardized outcome definitions and detailed reporting of intervention components.

## Limitations

This scoping review has some limitations that should be considered when interpreting the findings. First, this review did not search Embase despite searching multiple major databases, which may have resulted in the omission of relevant studies. This could have introduced selection bias. However, we attempted to mitigate this limitation by conducting comprehensive searches in PubMed/MEDLINE, the Cochrane Library, and the Cumulative Index to Nursing and Allied Health Literature Complete, supplemented with manual reference screening. Second, several included studies had small sample sizes or limited adherence to exercise protocols, which may have reduced statistical power and limited the ability to detect significant differences. Therefore, the true effects of exercise on treatment outcomes could be underestimated. Additionally, variability in exercise prescription and incomplete reporting of intervention details in some studies may affect the generalizability of these findings. Third, although the included studies provide valuable insights, the number of interventions addressing specific cancer types was limited, reflecting our outcome selection criteria rather than the availability of research in this field. Fourth, the studies included in this review were heterogeneous in terms of cancer type, treatment phase, exercise prescription, and outcome measures, which limits the ability to synthesize results quantitatively. However, this diversity highlights important considerations for designing future studies and tailoring interventions. Fifth, detailed information regarding exercise prescriptions, such as intensity, frequency, duration, and supervision, was not consistently reported. Additionally, several studies have provided incomplete information regarding key exercise prescription components, which we previously categorized as “unknown.” This limitation substantially compromises interpretability and reproducibility and constrains the ability to synthesize findings or draw meaningful conclusions regarding potential dose–response relationships. In particular, this review did not clarify the optimal intervention duration because many reports did not provide exact information on the exercise period. Additionally, it is anticipated that exercise frequency may decrease once treatment begins. Therefore, future studies should clearly define the intervention period a priori. Finally, by design, scoping reviews do not systematically assess study quality. Therefore, while many RCTs were included, we do not provide formal quality appraisal, and the strength of evidence for specific interventions may vary.

## Conclusions

This scoping review summarizes the current evidence on associations between exercise during chemotherapy or chemoradiotherapy and treatment delivery and tumor response outcomes in patients with cancer. Most studies focused on breast cancer, followed by colorectal cancer, and spanned various treatment phases, from neoadjuvant to adjuvant settings. Treatment-related outcomes most reflected treatment delivery and tolerance (e.g., relative dose intensity, dose delay or reduction, and treatment completion). Tumor response outcomes were reported, including pathological complete response, tumor size, response according to RECIST, and downstaging. Exercise interventions commonly included aerobic exercise alone or in combination with resistance training, were mostly supervised, and were often prescribed at moderate-to-high intensity, with a frequency of several times per week. Of the 31 interventions reviewed, nine demonstrated beneficial effects on treatment-related outcomes. However, the current evidence is insufficient to determine which specific exercise prescriptions are the most effective. Future RCTs should standardize and clearly report key exercise prescription variables, including intensity, timing, total training volume, progression, supervision, and adherence, to facilitate comparison and data pooling. Additionally, mechanistic studies are needed to clarify the relative contributions of fitness-mediated effects versus biology-mediated pathways, such as exerkine release and immune modulation, on treatment-related outcomes. Finally, future research should extend beyond breast and colorectal cancers. Addressing these priorities will facilitate the development of biologically informed and clinically actionable exercise interventions aimed at improving treatment-related outcomes in patients with cancer.

## Supplementary Information

Supplementary Material 1.

## Data Availability

All data generated or analyzed during this study are included in this published article. The authors have retained complete records of the extracted data and will make them available to the journal upon request.

## Abbreviations

IL: Interleukin
PRISMA-ScR: Preferred Reporting Items for Systematic Reviews and Meta-Analyses Extension for Scoping Reviews
RCT: Randomized controlled trials
RDI: Relative dose intensity
RECIST: Response Evaluation Criteria in Solid Tumors
SPARC: Secreted protein acidic and rich in cysteine
