# Supplementary material for: Exercise during chemotherapy or chemoradiotherapy and treatment delivery and tumor response outcomes: a scoping review
Source: BMC Cancer. 2026 Apr 24;26:723. doi: 10.1186/s12885-026-15992-6 (PMC13244998; doi:10.1186/s12885-026-15992-6)
Supplement: Supplementary file 1 — Supplementary Material 1. [file 12885_2026_15992_MOESM1_ESM.docx]

**Appendix 1**

**PubMed/MEDLINE**

Total number: 11969

| Search number | Query |
| --- | --- |
| 1 | "Neoplasms/therapy"[Mesh] |
| 2 | (((((malignan*[Title/Abstract]) OR (tumour*[Title/Abstract])) OR (tumor*[Title/Abstract])) OR (metastat*[Title/Abstract])) OR (carcinoma[Title/Abstract])) OR (neoplasm*[Title/Abstract]) |
| 3 | cancer treatmen*[Title/Abstract] |
| 4 | (((((((immunotherap*[Title/Abstract]) OR (neoadjuvant*[Title/Abstract])) OR (adjuvant*[Title/Abstract])) OR (hormonal therap*[Title/Abstract])) OR (chemotherap*[Title/Abstract])) OR (radiation[Title/Abstract])) OR (antitumor[Title/Abstract])) OR (anticancer[Title/Abstract]) |
| 5 | #1 OR #2 OR #3 OR #4 |
| 6 | treatment outcome[MeSH Terms] |
| 7 | ((((((((((((treatment efficac*[Title/Abstract]) OR (relative dose intensity[Title/Abstract])) OR (therapeutic effect*[Title/Abstract])) OR (completion rate*[Title/Abstract])) OR (tumor respons*[Title/Abstract])) OR (tumour respons*[Title/Abstract])) OR (treatment respons*[Title/Abstract])) OR (treatment efficac*[Title/Abstract])) OR (cancer outcom*[Title/Abstract])) OR (chemotherapeutic[Title/Abstract])) OR (radiotherapeutic[Title/Abstract])) OR (therapeutic[Title/Abstract])) OR (progressi*[Title/Abstract]) |
| 8 | #6 OR #7 |
| 9 | Exercise[MeSH Terms] OR Physical Therapy Modalities[MeSH Terms] |
| 10 | (exercis*[Title/Abstract] OR physical activit*[Title/Abstract] OR acute exercis*[Title/Abstract] OR isometric exercis*[Title/Abstract] OR aerobic*[Title/Abstract] OR exercise training*[Title/Abstract] OR walking*[Title/Abstract] OR resistance training*[Title/Abstract] OR cycling[Title/Abstract] OR bicycling[Title/Abstract] OR weight lifting[Title/Abstract] OR weight training[Title/Abstract] OR physiother*[Title/Abstract] OR physical therap*[Title/Abstract] ) OR (exercise therap*[Title/Abstract]) |
| 11 | (("Exercise Tolerance"[Mesh]) OR "Muscle Strength"[Mesh]) OR "Exercise Test"[Mesh] |
| 12 | ((((((((exercise tolerance[Title/Abstract]) OR (fitness test*[Title/Abstract])) OR (step test*[Title/Abstract])) OR (stress test*[Title/Abstract])) OR (treadmill test*[Title/Abstract])) OR (physical fitness test*[Title/Abstract])) OR (walk test*[Title/Abstract])) OR (muscle strength[Title/Abstract])) OR (arthrogenic muscle inhibition*[Title/Abstract]) |
| 13 | #9 OR #10 OR #11 OR #12 |
| 14 | #5 AND #8 AND #13 |
| 15 | English[Language] |
| 16 | (animals[MeSH Terms]) NOT (humans[MeSH Terms]) |
| 17 | (#14 AND #15) NOT #16 |

**Cochrane Library**

Total number: 4645

| ID | Search |
| --- | --- |
| #1 | MeSH descriptor: [Neoplasms] explode all trees and with qualifier(s): [therapy - TH] |
| #2 | (malignan*):ti,ab,kw OR (tumour*):ti,ab,kw OR (tumor*):ti,ab,kw OR (metastat*):ti,ab,kw OR (carcinoma):ti,ab,kw |
| #3 | (neoplasm*):ti,ab,kw OR (cancer NEXT treatmen*):ti,ab,kw OR (immunotherap*):ti,ab,kw OR (neoadjuvant*):ti,ab,kw OR (adjuvant*):ti,ab,kw |
| #4 | (hormonal NEXT therap*):ti,ab,kw OR (chemotherap*):ti,ab,kw OR (radiation):ti,ab,kw OR (antitumor):ti,ab,kw OR (anticancer):ti,ab,kw |
| #5 | #1 OR #2 OR #3 OR #4 |
| #6 | MeSH descriptor: [Treatment Outcome] explode all trees |
| #7 | (treatment NEXT efficac*):ti,ab,kw OR (relative NEXT dose NEXT intensity):ti,ab,kw OR (therapeutic NEXT effect*):ti,ab,kw OR (completion NEXT rate*):ti,ab,kw OR (tumor NEXT respons*):ti,ab,kw |
| #8 | (tumour NEXT respons*):ti,ab,kw OR (treatment NEXT respons*):ti,ab,kw OR (treatment NEXT efficac*):ti,ab,kw OR (cancer NEXT outcom*):ti,ab,kw OR (chemotherapeutic):ti,ab,kw |
| #9 | (radiotherapeutic):ti,ab,kw OR (therapeutic):ti,ab,kw OR (progressi*):ti,ab,kw |
| #10 | #6 OR #7 OR #8 OR #9 |
| #11 | MeSH descriptor: [Exercise] explode all trees |
| #12 | MeSH descriptor: [Physical Therapy Modalities] explode all trees |
| #13 | (exercis*):ti,ab,kw OR (physical NEXT activit*):ti,ab,kw OR (acute NEXT exercis*):ti,ab,kw OR (isometric NEXT exercis*):ti,ab,kw OR (aerobic*):ti,ab,kw |
| #14 | (exercise NEXT training*):ti,ab,kw OR (walking*):ti,ab,kw OR (resistance NEXT training*):ti,ab,kw OR (cycling):ti,ab,kw OR (bicycling):ti,ab,kw |
| #15 | (weight NEXT lifting):ti,ab,kw OR (weight NEXT training):ti,ab,kw OR (physiother*):ti,ab,kw OR (physical NEXT therap*):ti,ab,kw OR (exercise NEXT therap*):ti,ab,kw |
| #16 | MeSH descriptor: [Exercise Tolerance] explode all trees |
| #17 | MeSH descriptor: [Muscle Strength] explode all trees |
| #18 | MeSH descriptor: [Exercise Test] explode all trees |
| #19 | (exercise NEXT tolerance):ti,ab,kw OR (fitness NEXT test*):ti,ab,kw OR (step NEXT test*):ti,ab,kw OR (stress NEXT test*):ti,ab,kw OR (treadmill NEXT test*):ti,ab,kw |
| #20 | (physical NEXT fitness NEXT test*):ti,ab,kw OR (walk NEXT test*):ti,ab,kw OR (muscle NEXT strength):ti,ab,kw OR (arthrogenic NEXT muscle NEXT inhibition*):ti,ab,kw |
| #21 | {OR #11-#20} |
| #22 | #5 AND #10 AND #21 |

**CINAHL**

Total number: 2389

| **#** | Query |
| --- | --- |
| 1 | (MH "Neoplasms+/TH") |
| 2 | TI malignant OR TI tumors OR TI tumours OR TI metastatic OR TI carcinomas OR TI neoplasms OR TI cancer W0 treatments OR TI immunotherapy OR TI neoadjuvant OR TI adjuvant OR TI hormonal W0 therapy OR TI chemotherapy |
| 3 | AB malignant OR AB tumors OR AB tumours OR AB metastatic OR AB carcinomas OR AB neoplasms OR AB cancer W0 treatments OR AB immunotherapy OR AB neoadjuvant OR AB adjuvant OR AB hormonal W0 therapy OR AB chemotherapy |
| 4 | TI radiation OR TI antitumors OR TI anticancers |
| 5 | AB radiation OR AB antitumors OR AB anticancers |
| 6 | S1 OR S2 OR S3 OR S4 OR S5 |
| 7 | (MH "Treatment Outcomes+") |
| 8 | TI treatment W0 efficacy OR TI relative W0 dose W0 intensity OR TI therapeutic W0 effect OR TI completion W0 rate OR TI tumor W0 response OR TI tumour W0 response OR TI treatment W0 response OR TI treatment W0 efficacy OR TI cancer W0 outcoms OR TI chemotherapeutic OR TI radiotherapeutic OR TI therapeutic |
| 9 | AB treatment W0 efficacy OR AB relative W0 dose W0 intensity OR AB therapeutic W0 effect OR AB completion W0 rate OR AB tumor W0 response OR AB tumour W0 response OR AB treatment W0 response OR AB treatment W0 efficacy OR AB cancer W0 outcoms OR AB chemotherapeutic OR AB radiotherapeutic OR AB therapeutic |
| 10 | TI progressive OR AB progressive |
| 11 | S7 OR S8 OR S9 OR S10 |
| 12 | (MH "Exercise+") |
| 13 | (MH "Rehabilitation+") |
| 14 | TI exercise OR TI physical W0 activities OR TI acute W0 exercise OR TI isometric W0 exercise OR TI aerobic OR TI exercise W0 training OR TI walking OR TI resistance W0 training OR TI cycling OR TI bicycling OR TI weight W0 lifting OR TI weight W0 training |
| 15 | AB exercise OR AB physical W0 activities OR AB acute W0 exercise OR AB isometric W0 exercise OR AB aerobic OR AB exercise W0 training OR AB walking OR AB resistance W0 training OR AB cycling OR AB bicycling OR AB weight W0 lifting OR AB weight W0 training |
| 16 | TI physiotherapy OR TI physical W0 therapy OR TI exercise W0 therapy |
| 17 | AB physiotherapy OR AB physical W0 therapy OR AB exercise W0 therapy |
| 18 | S12 OR S13 OR S14 OR S15 OR S16 OR S17 |
| 19 | (MH "Exercise Tolerance+") |
| 20 | (MH "Muscle Strength+") |
| 21 | (MH "Exercise Test+") |
| 22 | TI exercise W0 tolerance OR TI fitness W0 test OR TI step W0 test OR TI stress W0 test OR TI treadmill W0 test OR TI physical W0 fitness W0 test OR TI walk W0 test OR TI muscle W0 strength OR TI arthrogenic W0 muscle |
| 23 | AB exercise W0 tolerance OR AB fitness W0 test OR AB step W0 test OR AB stress W0 test OR AB treadmill W0 test OR AB physical W0 fitness W0 test OR AB walk W0 test OR AB muscle W0 strength OR AB arthrogenic W0 muscle |
| 24 | S19 OR S20 OR S21 OR S22 OR S23 |
| 25 | S18 OR S24 |
| 26 | S6 AND S11 AND S25 |
